# Effect of Glass Ionomer Filler Size on Fluoride Release, Antiplaque Properties, and Abrasive Effects of Toothpaste

**DOI:** 10.1002/cre2.70109

**Published:** 2025-03-11

**Authors:** Behnaz Vahidi, Homayoon Alaghehmand, Hamed Tashakkorian, Seyedali Seyedmajidi, Maryam Ghasempour

**Affiliations:** ^1^ Student Research Committee Babol University of Medical Sciences Babol Iran; ^2^ Dental Materials Research Center, Health Research Institute Babol University of Medical Sciences Babol Iran; ^3^ Oral Health Research Center, Health Research Institute Babol University of Medical Sciences Babol Iran

**Keywords:** dental plaque, fluoride, glass ionomer, toothpaste

## Abstract

**Objectives:**

This study aimed to assess the impact of incorporating glass ionomer fillers of varying sizes on fluoride release, antiplaque properties, and the abrasive effects of toothpaste.

**Materials and Methods:**

In this laboratory study, three toothpaste samples containing glass ionomer filler particles (0.5, 5 µm, and a hybrid combination of equal proportions of both fillers) were tested, along with a base toothpaste without fillers or fluoride and a commercial toothpaste (Colgate Total) as controls. Fluoride release was measured using a fluoride ion‐selective electrode. Antiplaque properties were assessed by evaluating the effect of toothpaste on cultured saliva in tissue culture plates, with optical density measured using an ELISA reader. Abrasive effects were analyzed by changes in enamel thickness of bovine teeth using a three‐body wear test. Data were analyzed with ANOVA and Tukey's tests in SPSS software at a 0.05 significance level.

**Results:**

Toothpaste containing 0.5 µm fillers showed the highest fluoride release, whereas the lowest release was associated with 5 µm fillers. All formulations demonstrated antiplaque activity, though differences among filler‐containing toothpastes were not statistically significant (*p* > 0.05). Enamel abrasion differed significantly among the samples (*p* < 0.001), with the greatest abrasion observed for toothpaste containing 5 µm fillers and the least abrasion observed for 0.5 µm fillers.

**Conclusions:**

Incorporating glass ionomer fillers into toothpaste formulations provides fluoride release and antiplaque effects comparable to those of commercial toothpaste. Smaller filler sizes enhance fluoride release and reduce abrasiveness, though filler size does not significantly influence antiplaque efficacy.

## 1 Introduction

Preventive dentistry is a core principle of modern dental practice, focusing on minimally invasive treatments to preserve tooth structure and reduce patient discomfort (Hemagaran and Neelakantan 2014). There are various methods for managing dental caries, including controlling dietary sugar intake, practicing mechanical oral hygiene, using remineralization agents, and implementing preventive restorations (Featherstone 2004). Noninvasive care for early carious lesions (remineralization) has become a pivotal element in modern caries management guidelines (Amaechi 2015).

Remineralization is defined as the transport and deposition of mineral ions, predominantly calcium and phosphate, into the crystalline structure of demineralized dental tissue (Amaechi 2015). As the enamel lacks cellular repair capacity, salivary calcium and phosphate ions play a crucial role in facilitating enamel remineralization (Kanwal et al. 2018). This approach is especially important for reversing or halting the progression of early‐stage, noncavitated carious lesions through methods such as the use of fluoride‐containing toothpaste (Jefferies 2014; Kanwal et al. 2018).

Fluoride, the first recognized remineralizing agent, is regarded as the “gold standard” for caries prevention due to its unique mechanisms: reducing enamel demineralization, promoting remineralization, and inhibiting bacterial metabolism (Jabin et al. 2020; Philip 2019; Wiegand et al. 2007). Bioavailable fluoride (BF) refers to the free fluoride ions that are present in a soluble form within the toothpaste formulation. Total fluoride (TF) refers to the total amount of both soluble and insoluble fluoride, with the latter binding to abrasive materials. Fluoride must be bioavailable in toothpaste to effectively prevent tooth decay, that is, present as free, soluble fluoride ions accessible during brushing (Ko et al. 2015). The interaction of toothpaste abrasives with fluoride can reduce BF, potentially diminishing its anticaries efficacy (Reshetnyak et al. 2019).

Glass ionomer fillers, primarily silicate‐based compounds containing calcium and phosphate, provide key elements for remineralization in the oral environment (Omran et al. 2021). Research suggests that bioactive glass‐containing toothpastes can help alleviate dentinal hypersensitivity by forming hydroxyapatite layers that occlude dentinal tubules (Imran 2012; Tiskaya et al. 2021). These materials also demonstrate antibacterial properties by releasing ions in aqueous environments, which increase the pH and inhibit bacterial growth (Imran 2012). Despite these benefits, the abrasive effects of toothpaste containing fillers on the enamel and dentin must be carefully assessed. Toothpaste abrasiveness should effectively remove dental plaque and stains while avoiding damage to hard tissues (Hamza et al. 2022).

To date, limited studies have compared the effects of micron‐ and nano‐sized glass ionomer fillers on toothpaste properties. This study aims to examine the influence of filler size on fluoride release, antiplaque properties, and abrasiveness in toothpastes.

## 2 Materials and Methods

The study protocol was approved by the Ethics Committee of Babol University of Medical Sciences (IR.MUBABOL.HRI.REC.1402.288).

This laboratory‐based experimental study evaluated three toothpaste samples containing glass ionomer filler particles of 0.5, 5 µm, and a hybrid combination (equal proportions of 0.5 and 5 µm fillers), along with a base toothpaste without fillers or fluoride and a commercial toothpaste (Colgate Total) as controls.

The selection of 0.5 and 5 µm glass ionomer fillers was based on their representation of two distinct particle size ranges commonly used in dental materials: fine (nanoscale) and coarse (micron‐scale). These sizes were chosen based on the hypothesis of their differential effects on fluoride release, antiplaque properties, and abrasiveness, as they represent extremes in particle size distribution.

### 2.1 Preparation of Experimental Toothpastes

The components of the formulated toothpastes, their weight percentages, and roles are provided in Table 1. The base toothpaste was formulated by combining 14 mL of 70% sorbitol with 20 mL of water to create the liquid phase. Subsequently, 1.4 g of carboxymethylcellulose was mixed with 11.2 mL of glycerin and added to the liquid phase. Sodium benzoate (0.1 g) was incorporated, followed by 15 g of hydrated silica mixed with 16 mL of water. Lastly, 1.5 g of sodium lauryl sulfate was added gradually.

**Table 1:** Components of the formulated toothpastes, their weight percentages, and roles.

Ingredient	Manufacturer	Weight (%)	Function
Water	—	56	Diluent
Sorbitol	Chemex, Iran	9.8	Humectant
Sodium carboxymethylcellulose	Merck, Germany	1.4	Thickening agent
Glycerin	Merck, Germany	11.2	Humectant, thickener
Sodium benzoate	FIC, China	0.1	Preservative
Hydrated silica	FIC, China	15	Abrasive
Sodium lauryl sulfate	Sigma, USA	1.5	Detergent

The glass ionomer filler powder was synthesized by melting a mixture of silica, alumina, fluorite, cryolite, and calcium phosphate at 1250°C for 4 h. After the melting, the resulting mixture was cooled and subsequently, ground using a ball mill (PM100; Retsch Corporation, Germany) for 2 and 6 h to achieve particle sizes of 5 and 0.5 µm, respectively. The particle size of the filler was verified using Dynamic Light Scattering (Zetasizer, Malvern, UK), according to the method introduced previously (Marie 2021).

The classified filler particles, sized 0.5, 5 µm, and a hybrid (1:1 ratio of 0.5 and 5 µm), were then incorporated into the toothpaste base at a concentration of 5% by weight.

All ingredients were weighed using a precision balance (± 0.001 g, AND GR‐200, Japan) and mixed using a STEPHAN universal machine (UMC5 Mixer, Stephan Food Service Equipment GmbH, Germany).

The consistency of the toothpaste was adjusted by modifying the concentrations of carboxymethylcellulose and water to achieve a uniform texture. The samples were then stored at room temperature (20°–25°C) until testing.

### 2.2 Classification of Toothpaste Groups

The toothpastes were divided into five groups:

1. Test Group 1: Containing 0.5 µm glass ionomer fillers.
2. Test Group 2: Containing 5 µm glass ionomer fillers.
3. Test Group 3: Containing hybrid fillers (0.5 and 5 µm in equal proportions).
4. Control Group 1: Base toothpaste without fillers or fluoride.
5. Control Group 2: Commercial fluoride toothpaste (Colgate Total, Turkey; 1450 ppm fluoride), containing no glass ionomer fillers.

### 2.3 Measurement of Fluoride Release

For each toothpaste sample, both TF and BF concentrations were measured. The TF concentration was determined following the method outlined by the International Organization for Standardization (ISO 11609), whereas the BF concentration was assessed using the ISO TC106 standard. This method measures the soluble fluoride concentration in a toothpaste slurry after 1 min, corresponding to the estimated average duration of brushing.

- Sample Preparation
  First, 1 g of each toothpaste was mixed with 3 mL of 0.1 mol/L dipotassium phosphate (K₂HPO₄). Two slurry samples were prepared for each toothpaste: one for measuring TF and the other for measuring BF.
  ○ For the TF measurement, 1 mol/L hydrochloric acid (HCl) (Merck, Germany) was added to each test tube to ionize the fluoride. The samples were stored for 24 h and then centrifuged at 12,000 rpm for 10 min (Eppendorf, Germany). Then, 0.5 mL of the supernatant was transferred to a new microcentrifuge tube and mixed with an equal volume (0.5 mL) of total ionic strength adjustment buffer (TISAB) to prevent interference from other ions (Ko et al. 2015).
  Given the identical filler content in the toothpaste samples with fillers, the highest measured TF concentration among these three groups was reported as the TF value for all three groups.
  ○ For the BF measurement, the slurry was stirred for 1 min and then centrifuged at 12,000 rpm for 2 min. Next, 0.1 mL of the supernatant was mixed with 0.1 mL of 2 mol/L hydrochloric acid (HCl). The mixture was stored at room temperature for 24 h. After this period, 0.3 mL of TISAB solution was added to the sample, and the mixture was thoroughly homogenized (Ko et al. 2015).
- Fluoride Measurement
  • Finally, 4.95 mL of TISAB solution was added to 0.05 mL of each sample. Fluoride concentration was measured using a pH meter (Milwaukee MW150 MAX, Italy) equipped with a fluoride ion‐selective electrode (Sentek, UK). Calibration curves were generated using sodium fluoride standards. All experiments were performed in duplicate (Ko et al. 2015).

### 2.4 Evaluation of Toothpaste Abrasiveness

The abrasive effects of the toothpastes were examined on bovine enamel samples. Bovine teeth were chosen because they provide a consistent and readily available source of enamel with structural and compositional similarities to the human enamel, as widely documented in dental research. The minimum sample size for each group was calculated based on results from similar studies that compared the abrasive effects of toothpastes with different filler particle sizes (Mahmood et al. 2014). Considering a study accuracy of 95% (*α* = 0.05) and a power of 80% (*β* = 0.2), a minimum of 7 samples per group was determined, resulting in a total of 35 samples across the 5 groups.

- Sample Preparation
  First, the samples were examined under a stereomicroscope (Dewinter, Italy) at 40× magnification to ensure that they had intact enamel surfaces free from caries, cracks, discoloration, or other enamel defects. The samples were disinfected in a 0.2% thymol solution (Merck, Germany) for 24 h and then stored in physiological saline. The mounted teeth were sectioned using a three‐axis cutting machine (Nemo Fanavaran Pars, Mashhad, Iran) under water cooling to obtain 5 × 5 mm enamel blocks. The surfaces of the blocks were polished using silicon carbide sandpaper (KB, Turkey) with grit sizes ranging from 400 to 1200 for 60 s to achieve a smooth surface (Daryakenari et al. 2019).
- Measurement of enamel thickness

**Figure 1:**
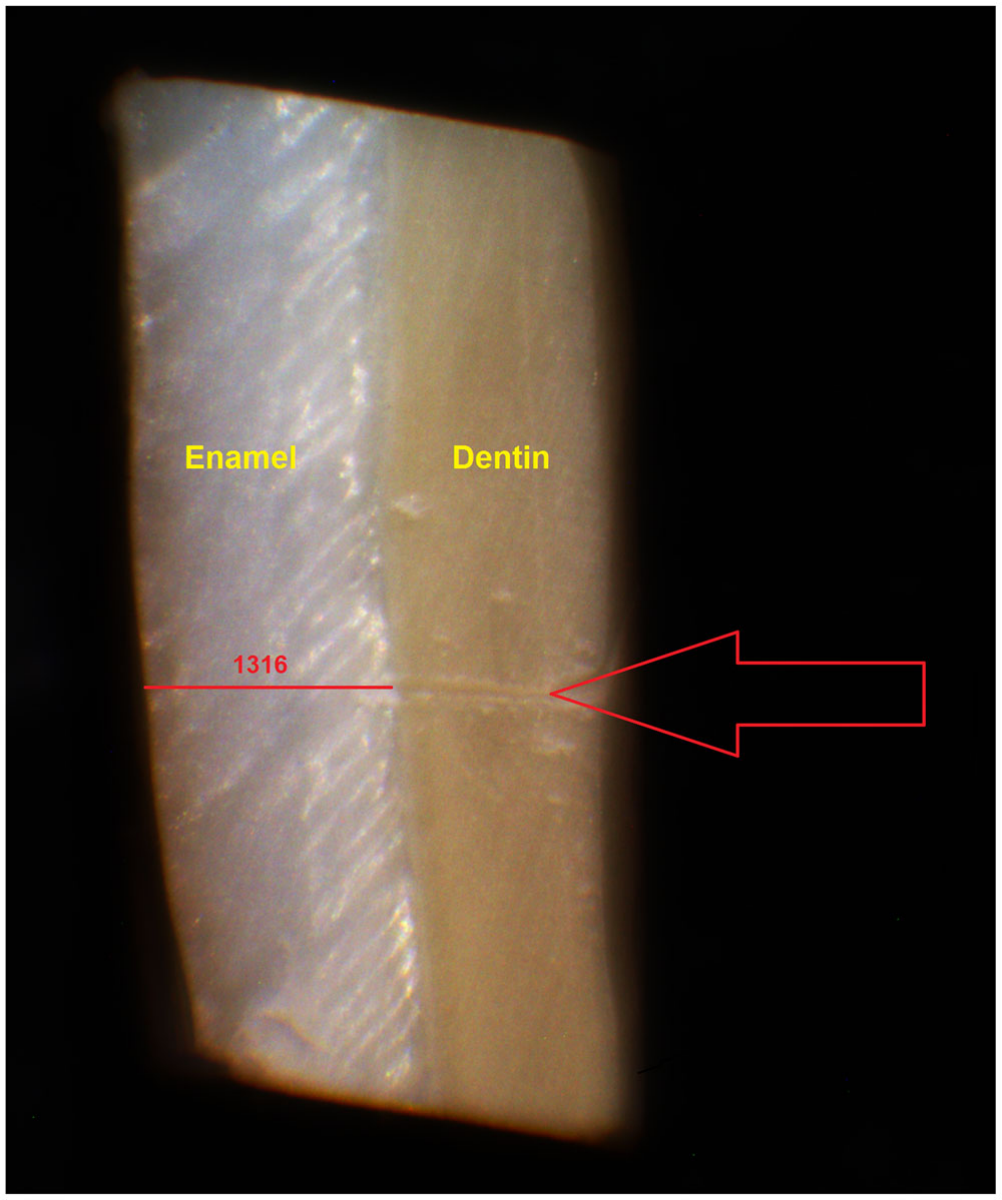
Measurement of enamel thickness. The enamel thickness was measured using Dewinter Capture Pro4.6 software. The red line shows the enamel thickness and the number above that shows the specific measurement related to the sample in micrometers. The red arrow indicates the reference point for measurement marked with a sharp bistoury.

The samples were imaged before abrasion from one of the lateral surfaces using a stereomicroscope (Dewinter, Italy) equipped with a 10‐megapixel camera at 70× magnification. The middle of the sample, marked with a sharp bistoury for consistency, was used as the reference point for measurement. The enamel thickness was measured using Dewinter Capture Pro4.6 software, with a precision of 1 µm. Each measurement was repeated three times, and the average value was calculated and recorded (Figure 1).

- Abrasion Test
  The abrasion process was performed using a three‐body wear method, involving toothpaste slurry, enamel, and a toothbrush (Medical 5138, soft, China) in a rotational motion with an automatic brushing device (PEDEB1, School of Dentistry, Babol, Iran). The toothpaste slurry consisted of 10 g of toothpaste, 40 mL of water, and 10 mL of 0.05% sodium carboxymethylcellulose (Merck, Germany). The brushing device was set to 0.25 bar pressure (200 gF) (Ledder et al. 2019) for 15,000 cycles (100 rpm for 150 min), which is approximately equivalent to 18 months of brushing (MoghareAbed et al. 2012). Based on the pilot study, the toothbrush was replaced after every 50 min to maintain consistent abrasion conditions.
- Remeasurement

After completing the abrasion process, the enamel blocks were washed and dried. Their thickness was then measured again using the same method as before, with a stereomicroscope. The difference in thickness represented the enamel wear. Each measurement was repeated three times (Daryakenari et al. 2019).

### 2.5 Antiplaque Property Analysis

To assess the antiplaque properties of the toothpastes, saliva samples were collected from 10 nonsmoking volunteers, each having at least 24 teeth, no periodontitis, and no systemic diseases or medications associated with such conditions. The mean age of the volunteers was 28.3 years (ranging from 20 to 40), with an equal gender distribution. The saliva samples were obtained at least 30 min after eating or drinking water. These individual saliva samples were subsequently pooled to create a cumulative sample, which was used for the experiments (Varma et al. 2018).

- Sample Preparation
  Two hundred microliters of saliva were added to each well in five rows of a 96‐well tissue culture plate (12 × 8) using a micropipette (ISOLAB, Germany). The plate was then incubated for 72 h at 37°C under aerobic conditions in an incubator (LTE Scientific Ltd, UK). After 72 h, the saliva was replaced with 100 µL of toothpaste slurry (1 g in 5 mL distilled water). The first row was left without toothpaste as the control. After 30 s, the slurry was aspirated. Next, 100 µL of the indicator erythrosine (Erythrosin B 1.5%, Sigma, USA) was added to each well, and was removed after 30 s. Finally, 100 µL of distilled water was added to all wells to dissolve the indicator (Singh et al. 2018).
- Optical density (OD) measurement

The OD was measured using an ELISA reader (BioTek Synergy HTX Multimode Plate Reader, Agilent Technologies, USA) at a wavelength of 540 nm (Singh et al. 2018). Higher absorbance indicated greater plaque presence and lower antiplaque efficacy.

### 2.6 Statistical Analysis

Data were analyzed using SPSS version 26. One‐way ANOVA and Tukey's post hoc tests were used to compare fluoride release, abrasion, and antiplaque properties among the groups. A significance level of 0.05 was used.

## 3 Results

### 3.1 Fluoride Release

The mean TF, BF, and the BF/TF ratio for the studied groups are shown in Table 2.

**Table 2:** Mean ± SD of total fluoride (TF), bioavailable fluoride (BF), and BF/TF ratio in the studied toothpaste.

Toothpaste	Toothpaste with 0.5 µm fillers	Toothpaste with 5 µm fillers	Toothpaste with hybrid fillers	Commercial toothpaste
Fluoride				
TF (ppm)	921 ± 2.19			1085 ± 14.24
BF (ppm)	641.5 ± 3.83	313 ± 2.19	333 ± 4.38	731.5 ± 11.5
BF/TF (%)	70	34	36	67

The mean BF levels were ranked as follows, from highest to lowest: commercial toothpaste, toothpaste with 0.5 µm filler, toothpaste with hybrid filler, and toothpaste with 5 µm filler. Among the filler‐containing toothpastes, the 0.5 µm filler formulation showed significantly higher BF levels compared to the other two formulations. Additionally, the BF/TF ratio for the toothpaste with 0.5 µm filler (70%) closely matched that of the commercial toothpaste (67%) and was about double the ratio observed in toothpastes containing 5 µm fillers or the hybrid filler.

### 3.2 Toothpaste Abrasiveness

The analysis of enamel thickness changes (wear) before and after abrasion revealed statistically significant differences in the mean wear values among the studied groups (*p* < 0.001). Table 3 presents the minimum, maximum, and mean wear values for each group. The lowest and highest average changes in enamel thickness were observed in the filler‐free toothpaste group and the commercial toothpaste group, respectively.

**Table 3:** Enamel thickness changes (in micrometers) in the studied groups.

Toothpaste	*N*	Mean ± SD	95% Confidence interval for mean		Min	Max	*p* value*
			Lower bound	Upper bound			
Toothpaste with 0.5 µm fillers	7	15 ± 5.35^A^	10.0483	19.9517	6.00	21.00	**< 0.001**
Toothpaste with 5 µm fillers	7	23.86 ± 7.42^a,b^	16.9894	30.7249	12.00	32.00	
Toothpaste with hybrid fillers	7	21 ± 6.58^b^	14.9119	27.0881	15.00	33.00	
Toothpaste without fillers	7	6.86 ± 3.24^B^	3.8637	9.8506	3.00	11.00	
Commercial toothpaste	7	28.57 ± 4.08^a,b^	24.8012	32.3417	21.00	33.00	

*Note:* Identical upper and lower case letters indicate statistically significant differences between groups based on Tukey's post hoc test.

* Analysis of variance (ANOVA) test.

The mean enamel thickness changes in the filler‐free toothpaste group were significantly lower than those in the other toothpaste groups (*p* < 0.001), except for the toothpaste containing 0.5 µm filler (*p* = 0.071). Among the three filler‐containing toothpaste groups, the toothpaste with 5 µm filler showed the highest mean wear, whereas the toothpaste with 0.5 µm filler showed the lowest mean wear. The difference in the mean wear between these two groups was statistically significant (*p* = 0.041). However, neither the 0.5 µm filler group nor the 5 µm filler group showed a statistically significant difference compared to the hybrid filler toothpaste group (*p* = 0.281 and *p* = 0.87, respectively).

The mean wear values in the hybrid filler toothpaste group were significantly different only when compared to the filler‐free toothpaste group (*p* < 0.001). The commercial toothpaste group showed significantly higher mean wear compared to the 0.5 µm filler toothpaste (*p* < 0.001) and the filler‐free toothpaste (*p* < 0.001), but no significant difference was observed when compared to the toothpastes containing 5 µm or hybrid fillers (*p* > 0.05).

### 3.3 Antiplaque Properties

**Figure 2:**
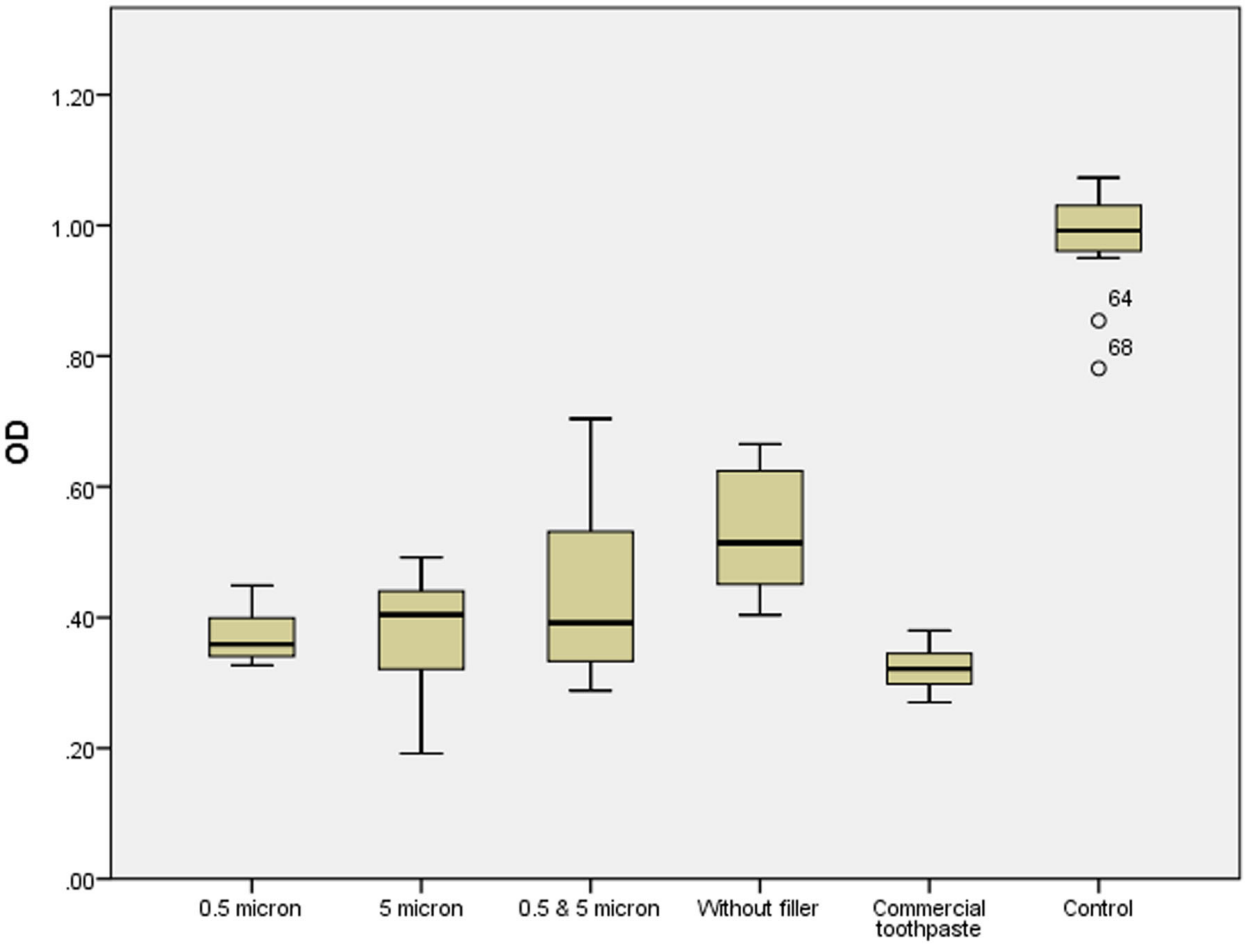
Comparison of the optical density (OD) of plaque in different toothpaste groups. The box plot displays the distribution of OD values, including the median (the line inside the box), the interquartile range (box), and outliers. The numbers 64 and 68 in the control group indicate sample IDs for outliers, which are data points falling outside the typical range of the data distribution.

The results related of the antiplaque effects of the toothpastes are presented in Figure 2. The mean OD values showed statistically significant differences between the studied groups (*p* < 0.001). The mean OD for the control group (0.98 ± 0.08) was significantly higher than that for the other groups (*p* < 0.001).

Among the five tested toothpastes, the lowest and highest mean OD values were observed in the commercial toothpaste (0.32 ± 0.03) and the filler‐free toothpaste (0.53 ± 0.1), respectively. The mean OD of the filler‐free toothpaste was significantly higher than that of the other toothpastes (*p* ≤ 0.001), except for the toothpaste containing the hybrid filler (0.43 ± 0.13) (*p* = 0.092).

Among the filler‐containing toothpastes, there were no statistically significant differences in the mean OD values (*p* > 0.05). The mean OD of the commercial toothpaste was significantly lower than that of the hybrid filler toothpaste and the filler‐free toothpaste, but it did not differ significantly from the toothpastes containing 0.5 µm (0.37 ± 0.04) and 5 µm (0.37 ± 0.09) fillers (*p* = 0.752 and *p* = 0.699, respectively).

## 4 Discussion

The present study investigated the effect of filler size on fluoride release, antiplaque properties, and abrasive effects in three experimental toothpastes containing glass ionomer fillers with particle sizes of 0.5, 5 µm, and a hybrid combination (equal proportions of 0.5 and 5 µm particles). The findings revealed that fluoride release and abrasiveness were influenced by filler size, whereas antiplaque properties remained largely unaffected.

### 4.1 Fluoride Release

The findings showed that fluoride release, particularly BF, was the highest in the commercial toothpaste group, followed by the toothpaste with 0.5 µm fillers, and the lowest in the toothpaste with 5 µm fillers. This suggests that smaller filler sizes enhance fluoride release in toothpaste formulations. Although previous studies have explored fluoride release from bioactive glass‐containing toothpastes, few have specifically examined the impact of filler size on fluoride availability.

Hirose et al. reported similar findings, demonstrating that salivary fluoride concentrations remained elevated after brushing with toothpastes containing 5% surface pre‐reacted glass ionomer (S‐PRG) fillers with particle sizes of 1 µm. Their study revealed that fluoride concentrations in saliva after the use of this toothpaste were comparable to those of a toothpaste containing aminofluoride, even 180 min post‐brushing. Moreover, these concentrations were higher than those observed with toothpastes containing sodium fluoride or monofluorophosphate. The authors also noted that there was no significant difference in fluoride concentration between toothpastes containing 5% and 10% glass ionomer fillers (Hirose et al. 2023). Although the formulations in their study were similar to those in the current investigation, it is important to note that Hirose et al.'s study was conducted in an in vivo oral environment, whereas the present study utilized different methodological conditions.

In the present study, the BF values were substantially lower than the TF levels in all toothpastes. Specifically, BF accounted for approximately two‐thirds of the TF in the toothpaste containing 0.5 µm fillers and the commercial toothpaste, whereas it represented about one‐third in the toothpaste with 5 µm fillers and the hybrid formulation. This discrepancy is attributed to the fluoride being bound to the filler components.

Oliveira et al. (2013) similarly observed that the fluoride available from fluoride‐containing toothpastes in children was consistently lower than the TF content in the toothpaste. Shen et al. (2021) found that the BF in six commercial fluoride‐containing toothpastes varied between 27% and 97% of the TF content. In the present study, the percentage of BF relative to TF ranged from 34% to 70%. This variation compared to other studies may be attributed to differences in toothpaste formulations. The toothpastes studied by Shen et al. contained calcium phosphate compounds along with sodium fluoride or monofluorophosphate. Only in one formulation, which included casein phosphopeptide‐amorphous calcium phosphate and sodium fluoride, was the percentage of BF nearly identical to the TF content (97%). In contrast, other toothpastes containing calcium phosphate compounds showed much lower BF levels (Shen et al. 2021). The bioavailability of fluoride in toothpastes is influenced by the type of fluoride salt used as well as the abrasive components in the formulation. For instance, due to the incompatibility of sodium fluoride, stannous fluoride, and even aminofluoride with calcium carbonate abrasives, silica particles are commonly used in conjunction with these fluoride salts to enhance fluoride solubility and ensure its efficacy in preventing dental decay (Oliveira et al. 2013).

Although fluoride is widely recognized for its benefits in enamel remineralization and caries prevention, excessive ingestion, especially in children under 4 years of age who may not rinse properly, can pose risks. Recent studies highlight that high doses of systemic fluoride can cause dentine dysplasia (Okamoto et al. 2025). This is particularly concerning, given that humans are more sensitive to fluoride than experimental models like mice. Therefore, although our study demonstrates the advantages of fluoride‐releasing glass ionomer fillers in toothpaste formulations, it is crucial to consider the potential risks of fluoride toxicity in vulnerable populations. This underscores the importance of cautious use and parental supervision when administering fluoride‐containing products to young children.

Consistent with the findings of Ko et al. the TF measured in the commercial toothpaste group in the present study was lower than the TF content reported by the manufacturer. They measured the TF in six commercial toothpastes containing different fluoride compounds and abrasives, and found that the actual fluoride content was consistently lower than the claimed amount, with measured values ranging from 53% to 93% of the stated fluoride content (Ko et al. 2015). This discrepancy could be attributed to interactions between the fluoride compounds and abrasive particles in the toothpaste formulation. The fluoride measurement method used in their study was similar to that used in the present study.

In contrast, Shen et al. (2021) found that the TF concentration in all the commercial toothpastes that they analyzed was either close to or very close to the TF content indicated on the product labels (ranging from 94% to 105%). This difference may be explained by the distinct fluoride measurement techniques used in the two studies. In the present study, fluoride concentration was measured using a fluoride ion‐selective electrode, a method with lower technical sensitivity but high measurement accuracy. However, components such as detergents and humectants in the toothpaste may contaminate the electrode, potentially interfering with the fluoride ion measurements. On the other hand, Shen et al. (2021) used the microdiffusion method for fluoride measurement, which allows for accurate fluoride detection without the risk of electrode contamination.

### 4.2 Abrasive Effects

In the present study, the average abrasion values for all three glass‐ionomer filler‐containing toothpastes ranged from 15 to 25 µm, corresponding to a brushing duration equivalent to 18 months with a brushing force of 200 g. As a result, concerns regarding the abrasion caused by toothpastes containing glass‐ionomer fillers appear to be minimal. The highest average abrasion was observed in the toothpaste containing 5 µm fillers, which was significantly greater than that of the toothpaste containing 0.5 µm fillers. This suggests that the particle size of the filler influences the abrasive properties of the toothpaste, with larger filler particles leading to increased abrasion. On the other hand, although smaller particle sizes incur higher production costs, there are also limitations associated with the use of smaller filler particles.

The results of the present study were consistent with those of Mahmood et al. who demonstrated that an increase in filler particle size could lead to greater enamel abrasion. However, the abrasion values reported in their study, measured in micrometers, were higher than those in the present study. This discrepancy may be attributed to differences in the composition and particle size of the glass fillers used in their study, which ranged from 38 to 110 µm—substantially larger than those used in the present study. Additionally, the toothpaste formulations in Mahmood et al.'s study contained abrasive silica particles, whereas the present study utilized hydrated silica particles. Furthermore, the effect of the shape of the glass‐ionomer filler particles on enamel abrasion was also explored in their study, which found that angular and irregularly shaped particles caused more abrasion than spherical particles (Mahmood et al. 2014).

Toothpastes contain a variety of abrasive agents, including hydrated silica, alumina, dicalcium phosphate dihydrate, insoluble metaphosphate, and calcium carbonate (Macdonald et al. 2010). Among these, silica derivatives are the most commonly used due to their effectiveness in removing bacterial plaque and preventing the buildup of other superficial deposits on tooth surfaces (Aguiar et al. 2017). The abrasiveness of toothpaste is influenced by several factors, such as the material, microstructure, concentration, size, and shape of the abrasives (Bruno et al. 2016). Colgate Total contains hydrated silica, which consists of mineral compounds with varying physicochemical properties (da Rosa et al. 2016). Its highly abrasive nature contributes to its exceptional cleaning ability, making it the most abrasive among the products investigated in the present study.

It is evident that the actual abrasion values in the oral environment will differ from those measured under laboratory conditions. Factors such as exposure to acidic challenges immediately before brushing can lead to increased enamel abrasion (Mahmood et al. 2014). Conversely, the presence of an acquired pellicle and plaque on the tooth surface may offer a protective effect against abrasion caused by toothpaste. Given the lack of fixed reference points within the oral cavity, intraoral measurement of abrasion values is not feasible. Therefore, although laboratory studies may not provide an exact estimate of abrasion caused by toothpastes in the oral environment, they can still serve as a useful tool for comparing the abrasion potential of different toothpastes (MoghareAbed et al. 2012).

### 4.3 Antiplaque Properties

Based on the results of the present study, the greatest plaque‐inhibiting effect was observed in the commercial toothpaste, followed by the glass‐ionomer filler‐containing toothpastes with 0.5 and 5 µm particle sizes, with no statistically significant difference between them. However, the plaque‐inhibiting effect of the toothpaste containing the hybrid filler was significantly lower than that of the commercial toothpaste. The plaque‐inhibiting effects of the toothpaste formulations containing glass‐ionomer fillers did not show any significant statistical difference. Thus, it can be concluded that the addition of glass‐ionomer filler particles to toothpaste can result in plaque‐inhibiting effects, but the particle size of the glass‐ionomer filler does not have a significant impact on these effects.

In agreement with the findings of the present study, Silva et al. demonstrated that toothpastes containing varying weight percentages of glass‐ionomer fillers can exert inhibitory effects on dental plaque microorganisms. They attributed the observed plaque‐inhibiting effects to the ability of these toothpastes to release various ions, including borate, strontium, and fluoride (da Silva Spinola et al. 2023). Additionally, the study by Kono et al. (2021) showed that the ions released from glass‐ionomer fillers used in toothpaste formulations, including fluoride, aluminum, silica, strontium, and borate ions, can induce oxidative stress in plaque‐forming microorganisms and exert inhibitory effects on them.

### 4.4 Clinical Implications and Limitations

The findings suggest that toothpaste containing 0.5 µm fillers may provide the best balance between enhanced fluoride release and reduced enamel abrasiveness. However, larger fillers may offer prolonged ion release and improved remineralization over time, which warrants further investigation (Mahmood et al. 2014).

This study has some limitations. The experimental conditions do not fully replicate the oral environment, where factors such as salivary flow, plaque biofilm, and dietary habits may influence fluoride release and abrasiveness. Future in vivo studies are needed to confirm these results.

## 5 Conclusion

Toothpastes containing glass ionomer fillers demonstrated the capability to release fluoride and showed antiplaque properties comparable to those of commercial fluoride toothpaste. The size of the glass ionomer fillers influenced both fluoride release and abrasiveness. Smaller filler sizes resulted in higher BF release and lower enamel abrasion, making them a potentially advantageous choice for toothpaste formulations. However, filler size had no statistically significant impact on antiplaque efficacy.

These findings suggest that incorporating glass ionomer fillers, particularly at smaller sizes, may improve the therapeutic benefits of fluoride toothpaste while minimizing enamel wear. Further clinical studies are recommended to evaluate the long‐term effects and potential remineralization benefits of such formulations.

## Author Contributions

Behnaz Vahidi conducted the project work, collected data, and drafted the manuscript. Homayoon Alaghehmand contributed to conceptualization and methodology. Hamed Tashakkorian contributed to chemical advising and methodology. Seyedali Seyedmajidi contributed to formal data analysis and editing of the manuscript. Maryam Ghasempour supervised the project and revised the manuscript. All authors have read and approved the final manuscript.

## Ethics Statement

The study protocol was approved by the Ethics Committee of Babol University of Medical Sciences (IR.MUBABOL.HRI.REC.1402.288).

## Conflicts of Interest

The authors declare no conflicts of interest.

## Data Availability

The data are available upon reasonable request from the corresponding author.
